# Overexpression and Characterization of a Novel Thermostable β-Agarase YM01-3, from Marine Bacterium *Catenovulum agarivorans* YM01^T^

**DOI:** 10.3390/md12052731

**Published:** 2014-05-12

**Authors:** Fangyuan Cui, Sujie Dong, Xiaochong Shi, Xia Zhao, Xiao-Hua Zhang

**Affiliations:** 1 College of Marine Life Sciences, Ocean University of China, Qingdao 266003, China; E-Mails: joyceyujian@163.com (F.C.); dongsujie310@163.com (S.D.); xhzhang@ouc.edu.cn (X.-H.Z.); 2 School of Medicine and Pharmacy, Ocean University of China, Qingdao 266003, China; E-Mail: zhaoxia@ouc.edu.cn

**Keywords:** *Catenovulum agarivorans* YM01^T^, expression, characterization, YM01-3, β-agarase, thermostable

## Abstract

Genome sequencing of *Catenovulum agarivorans* YM01^T^ reveals 15 open-reading frames (ORFs) encoding various agarases. In this study, extracellular proteins of YM01^T^ were precipitated by ammonium sulfate and separated by one-dimensional gel electrophoresis. The results of in-gel agarase activity assay and mass spectrometry analysis revealed that the protein, YM01-3, was an agarase with the most evident agarolytic activity. Agarase YM01-3, encoded by the YM01-3 gene, consisted of 420 amino acids with a calculated molecular mass of 46.9 kDa and contained a glycoside hydrolase family 16 β-agarase module followed by a RICIN superfamily in the C-terminal region. The YM01-3 gene was cloned and expressed in *Escherichia coli*. The recombinant agarase, YM01-3, showed optimum activity at pH 6.0 and 60 °C and had a *K*_m_ of 3.78 mg mL^−1^ for agarose and a *V*_max_ of 1.14 × 10^4^ U mg^−1^. YM01-3 hydrolyzed the β-1,4-glycosidic linkages of agarose, yielding neoagarotetraose and neoagarohexaose as the main products. Notably, YM01-3 was stable below 50 °C and retained 13% activity after incubation at 80 °C for 1 h, characteristics much different from other agarases. The present study highlights a thermostable agarase with great potential application value in industrial production.

## 1. Introduction

Agar, as one of the marine polysaccharides used widely at present, is a component found in the cell walls of some marine red algae, *Gracilariales* and *Gelidiales*, and consists of two different components, agarose and agaropectin [1]. Agarose is a neutral linear polysaccharide composed of alternating residues of 3-*O*-linked β-d-galactopyranose and 4-*O*-linked 3,6-anhydro-α-l-galactopyranose [2]. Agaropectin has the same basic disaccharide-repeating units as agarose, although some hydroxyl groups of 3,6-anhydro-α-l-galactose residues are substituted by sulfoxy or methoxy and pyruvate residues.

Agarases are a group of glycoside hydrolases (GH) that degrade agar into oligosaccharides. According to the cleaving mechanisms, agarases could be classified into two groups, *i.e.*, α-agarases (EC 3.2.1.158), which cleave α-1,3 linkages to produce agaro-oligosaccharides [3], and β-agarases (EC 3.2.1.81), which cleave β-1,4 linkages to produce neoagarooligosaccharides [4]. According to the CAZy database [5,6], β-agarases are classified into four families of GH16, GH50, GH86 and GH118, based on the amino acid sequence similarity [7]. To date, many bacterial genera with agarolytic activity have been identified, which were mainly isolated from seawater and marine sediment [8], including the genera, *Agarivorans* [9], *Alteromonas* [10], *Microscilla* [11], *Pseudoalteromonas* [12], *Pseudomonas* [13], *Pseudozobellia* [14], *Saccharophagus* [15], *Vibrio* [16], *etc.* In addition, there are a few agarolytic bacterial genera from freshwater and terrestrial soils, such as *Acinetobacter* [17], *Bacillus* [18], *Cellvibrio* [8] and *Cytophaga* [19]. Agarases produced by the above bacteria are potential biocatalysts to modify agars and alter their properties, producing new, specific algal biomolecules for foods, cosmetics and pharmaceuticals [20]. *Catenovulum agarivorans* YM01^T^, an agar-hydrolyzing marine bacterium, recently isolated from seawater of the Yellow Sea of China, was identified as a novel genus and species [21]. It has been shown that the extracellular proteins of YM01^T^ have high agarolytic activity and thermostability. Moreover, 15 complete coding sequences of agarases (including two α-agarases and 13 β-agarases) from the *C*. *agarivorans* YM01^T^ genome were identified by the whole-genome sequencing [22]. In this study, to explore the characteristics and commercial significance of *C*. *agarivorans* agarase, the β-agarase gene, YM01-3, with the most evident agarolytic activity according to the in-gel agarase activity assay and mass spectrometry analysis was cloned and overexpressed. The purified recombinant agarase was characterized, and its enzymatic products were analyzed. On account of its noticeable high agarolytic activity and thermostability, this enzyme has potential industrial applications.

## 2. Results and Discussion

### 2.1. Agarolytic Activity of the Extracellular Proteins of YM01^T^

**Figure 1:**
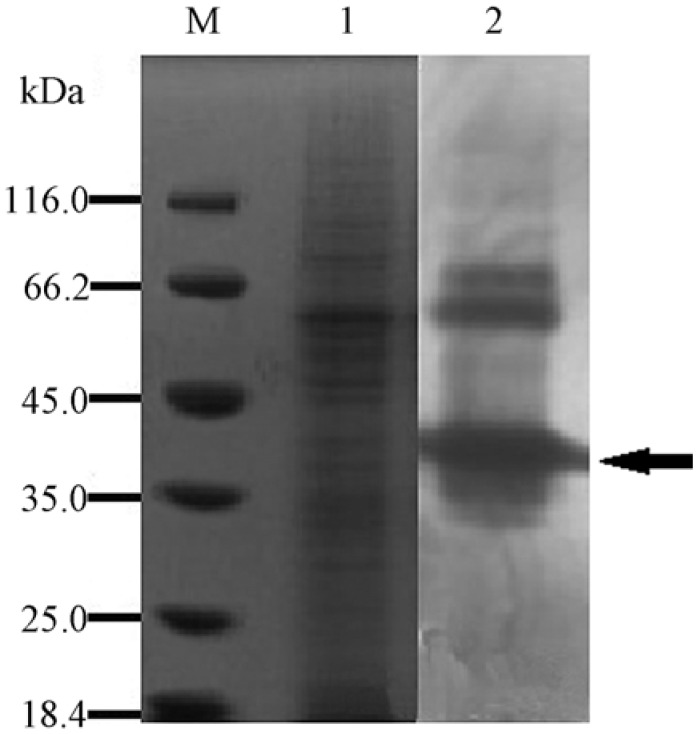
SDS-PAGE and *in situ* detection of agarolytic activity. Lane M, molecular mass marker (Fermentas SW0431); Lane 1, the crude extracellular proteins of YM01^T^; Lane 2, *in situ* detection of the crude agarase, and the arrow indicates the band cut for MS analysis. SDS-PAGE was performed with 10% (w/v) polyacrylamide gel. The gel with Lane M and Lane 1 was stained with Coomassie brilliant blue R-250; the agar plate used for in-gel detection (Lane 2) was stained by Lugol’s iodine solution.

The result of SDS-PAGE and in-gel detection of agarase activity (Figure 1) showed that the extracellular proteins of YM01^T^ had agarolytic activity, and the protein band of approximately 40 kDa on SDS-PAGE showed the most evident agarase activity. The result of mass spectrometry analysis revealed that the 40-kDa protein band contained the YM01-3 gene encoded agarase along with several other proteins, e.g., lipoprotein, flagellin and aspartate-semialdehyde dehydrogenase. The YM01-3 gene (GenBank accession No. KF413621) was therefore chosen for further study.

### 2.2. Cloning and Sequence Analysis of the YM01-3 Gene

**Figure 2:**
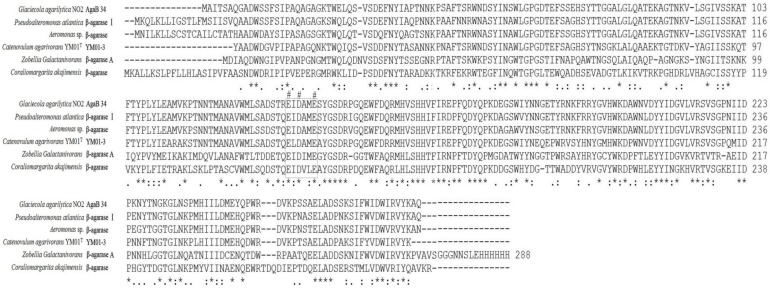
Multiple amino acid sequence alignment of the YM01-3 agarase of *Catenovulum agarivorans* YM01^T^ with known GH16 family β-agarases. Identical residues in all sequences are indicated by asterisks under the column; conserved substitutions are indicated by colons, and semi-conserved substitutions are indicated by dots; pound signs highlight the catalytic residues, and black rectangles highlight the catalytic motif. Deletions are indicated by dashes.

**Figure 3:**
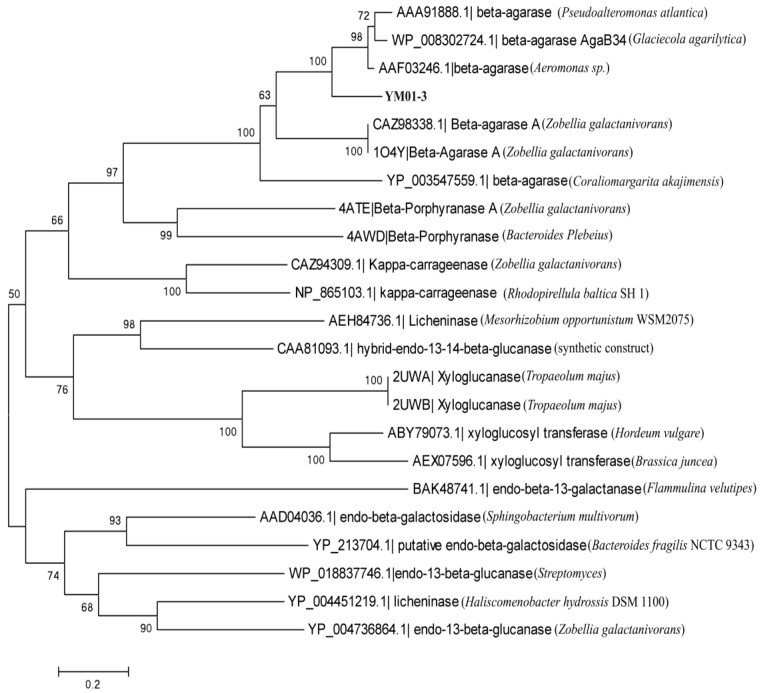
Neighbor-joining tree based on amino acid sequences of YM01-3 and other GH16 family members showing the relationship among protein YM01-3 and other known glycoside hydrolase from GH16. Numbers at nodes are the levels of bootstrap support (%). Scale bar, 0.2 substitutions per amino acid position.

The YM01-3 gene, a 1263-bp open reading frame (ORF), was amplified from the genomic DNA of *C*. *agarivorans* YM01^T^ using the primer sets. The ORF encoded a deduced protein of 420 amino acids, with an estimated molecular mass of 46.9 kDa and a putative isoelectric point of 5.66. No putative signal peptide was detected with the SignalP 4.0 server [23]. Sequence analysis using BLAST search in the database, NCBI, showed that the deduced protein was mainly comprised of a 269 amino acid β-agarase domain that was homologous to the catalytic module of family 16 glycoside hydrolase (GH16) and a 134 amino acid sequence belonging to the RICIN superfamily (ricin-type beta-trefoil; carbohydrate-binding domain formed from presumed gene triplication). The protein displayed the catalytic residues (Glu^130^-Asp^132^-Glu^135^) and the catalytic motif (E[ILV]D[IVAF][VILMF]_[0,1]_E), which are prevalent in the agarases belonging to the GH16 family. Glu^130^ and Glu^135^ act as nucleophile and an acid/base, respectively, while Asp^132^ is probably important in maintaining the charges in the environment of catalytic amino acids [24]. Moreover, the encoded protein showed high identity to other β-agarases of the GH16 family: 76% to the β-agarase AgaB34 from *Glaciecola agarilytica* NO2 (WP_008302724.1), 76% to the β-agarase I from *Pseudoalteromonas atlantica* (AAA91888), 74% to the β-agarase from *Aeromonas* sp. (AAF03246), 56% to the β-agarase A from *Zobellia galactanivorans* (1O4Y) and 52% to the β-agarase from *Coraliomargarita akajimensis* DSM 45221 (YP_003547559.1) (Figure 2). The GH16 family, consisting of more than 2700 members, is classified into ten specific subfamilies, including β-agarase (EC 3.2.1.81), licheninase (EC 3.2.1.73), κ-carrageenase (EC 3.2.1.83), xyloglucanase (EC 3.2.1.151), endo-β-1,3-galactanase (EC 3.2.1.181), β-porphyranase (EC 3.2.1.178), *etc.* [6,25]. To determine the relationship between the YM01-3 protein and other known glycoside hydrolases from GH16, a neighbor-joining tree (Figure 3) based on amino acid sequences was constructed, which revealed the evolutionary relationship between protein YM01-3 and other known glycoside hydrolases and showed that YM01-3 formed a tight phylogenetic cluster in the β-agarase clade.

### 2.3. Expression and Purification of the Recombinant YM01-3

**Figure 4:**
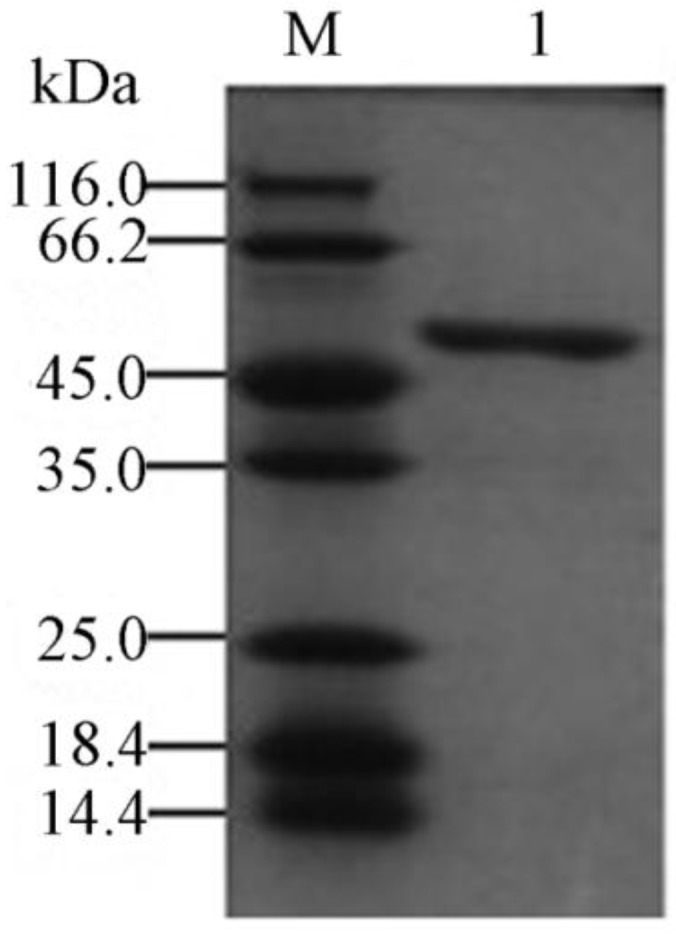
SDS-PAGE of purified recombinant agarase YM01-3. Lane M, molecular mass marker (Fermentas SW0431); Lane 1, purified agarase YM01-3. SDS-PAGE was performed with 12% (w/v) polyacrylamide gel. Gels were stained with coomassie brilliant blue R-250.

**Figure 5:**
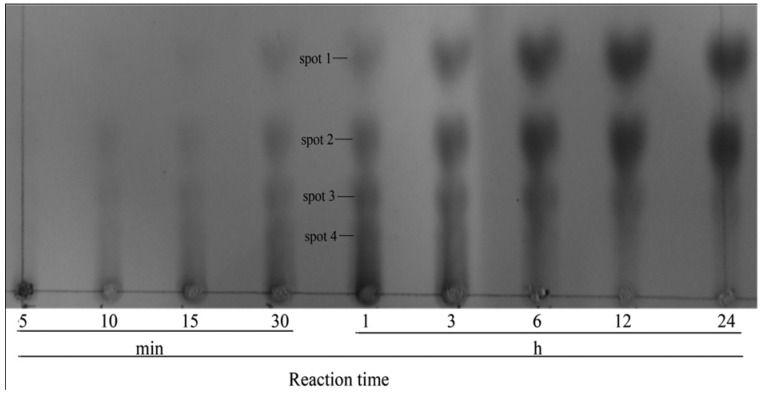
TLC chromatogram depending on the reaction time. The hydrolysis reactions were carried out at 50 °C for 24 h in 20 mM Tris-HCl buffer (pH 8.0) containing 0.25% agarose and then separated on a Silica Gel 60 TLC plate at appropriate time intervals.

The recombinant plasmid pET-24a (+)/YM01-3 was conditionally expressed in the *E*. *coli* BL21 (DE3) as a *C*-terminally His-tagged recombinant protein. After IPTG (isopropyl-β-d-thiogalactopyranoside) induction, the agarolytic activity of the recombinant protein was detected in the supernatant of the cell lysate. To maintain its bioactivity, the recombinant protein was purified from the supernatant under native conditions. The purified enzyme (including the 6-His tag) gave a single band with a molecular weight of about 46 kDa on SDS-PAGE (Figure 4). Further analysis by mass spectroscopy showed that the accurate molecular mass of YM01-3 (including the histidine tag and 16 amino acids from pET 24a (+)) was 49.528 kDa [26]. To find out whether agarase YM01-3 was an endo- or exo-glycoside hydrolase, as well as to determine its end hydrolysis products, a time course hydrolysis analysis was carried out with the purified recombinant enzyme. As shown in Figure 5, four major spots were clearly detected. Ion trap mass spectra analysis of the Spot 1 revealed that it has a molecular ion at m/z of 629.19 [M − H]^−^ and 651.17 [M + Na − 2H]^−^, and it was determined to be neoagarotetraose (DP4). Meanwhile, Spot 2, Spot 3 and Spot 4 were assigned as neoagarohexaose (DP6) (*m*/*z* = 935.29 [M − H]^−^, 997.29 [M + HCO_3_]^−^), neoagarooctaose (DP8; *m/z* = 1241.37 [M − H]^−^, 1303.37 [M + HCO_3_]^−^, 1325.35 [M + HCO_3_ + Na − 2H]^−^, 620.18 [M − 2H]^2−^) and neoagarodecaose (DP10; *m*/*z* = 1547.47 [M − H]^−^, 1609.47 [M + HCO_3_]^−^, 1631.45 [M + HCO_3_ + Na − 2H]^−^, 773.23 [M − 2H]^2−^), respectively. According to the results of thin-layer chromatography (TLC) and MS analysis, agarose incubation with YM01-3 generated DP10 and DP8 firstly, followed by DP6 and DP4, and the amount of DP6 and DP4 increased in a time-dependent manner. This hydrolysis pattern indicated that β-agarase YM01-3 was an endo-type β-agarase. In addition, after incubation for 24 h, the main products were DP6 and DP4, which is a characteristic of the GH16 family agarases. Therefore, with the evidence obtained above, we conclude that the β-agarase YM01-3 should be cataloged into family 16 of glycoside hydrolases and proceeded with a common catalytic mechanism of GH16 family, which led to the overall retention of the anomeric configuration and to *trans*-glycosylating activity [25].

### 2.4. Biochemical Characterization of YM01-3

**Figure 6:**
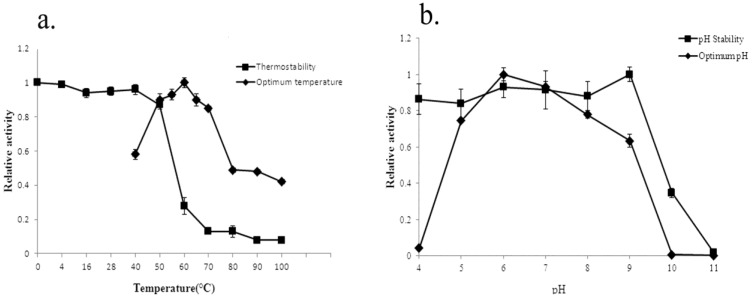
Effect of temperature (**a**) and pH (**b**) on the agarolytic activity and stability of the recombinant β-agarase YM01-3. Temperature profiles were measured at different temperatures (0–100 °C) in 20 mM Tris-HCl buffer (pH 8.0). pH profiles were measured at 50 °C in different buffers. All data shown are the mean values from at least three replicate experiments.

Both the effects of temperature and pH on the activity of the recombinant agarase were evaluated. The results showed that the optimal temperature for β-agarase YM01-3 was 60 °C (Figure 6a), and YM01-3 retained more than 80% agarolytic activity after being kept in 50 °C for 1 h, 13% residual activity after incubation in 80 °C for 1 h (Figure 6a) and 12% residual activity, even if being boiled for 5 min [26]. The enzyme exhibited maximum agarase activity at pH 6.0 and retained more than 80% of activity after incubation at a wide range of 4.0–9.0 for 12 h at 4 °C (Figure 6b).

The results of the effect of various metal ions and agents on the activity of the purified agarase (Table 1) showed that some metal ions, such as Cu^2+^, Mn^2+^, Fe^3+^ and Ni^2+^, had a highly significant negative effect on the activity of the agarase, YM01-3, while Na^+^, K^+^ and Ca^2+^ increased the agarase activity significantly. In addition, Mg^2+^, urea, EDTA and SDS did not have obvious effects on the activity.

Based on the results obtained above, all enzymatic reactions were performed in citric acid-sodium citrate buffer (pH 6.0) at 60 °C, the *K*_m_ and *V*_max_ for agarose were 3.78 mg mL^−1^ and 1.14 × 10^4^ U mg^−1^ of protein, respectively. Compared the kinetic parameters of YM01-3 to those of other known β-agarases, the *V*_max_ value (1.14 × 10^4^ U mg^−1^) was remarkably higher than that of agarase AgaA (909.1 U mg^−1^) [27], which is the highest *V*_max_ value reported (Table 2).

**Table 1:** Effects of various reagents on the activity of the recombinant β-agarase YM01-3.

Reagent	Concentration (mM) ^a^	Relative Activity (%) ^b^
None		100
SDS	1	109 ± 0.02
Urea	1	101 ± 0.056
EDTA	1	81 ± 0.016
Na^+^	1	122 ± 0.13 **
K^+^	1	122 ± 0.12 **
Ca^2+^	1	113 ± 0.13 *
Mg^2+^	1	88 ± 0.07
Mn^2+^	1	28 ± 0.04 **
Fe^3+^	1	14 ± 0.03 **
Cu^2+^	1	34 ± 0.08 **
Ni^2+^	10	1 ± 0.01 **

The activity was analyzed with 0.25% (w/v) agarose in 20 mM Tris-HCl buffer (pH 8.0) at 50 °C for 10 min. Results are shown as the mean ± SEM from three independent experiments. ^a^ The concentration is the final concentration of the reagents in the activity detection system; ^b^ the activity measured under the condition without being treated with various ions and reagents was defined as 100%; * *p* < 0.05; ** *p* < 0.01.

**Table 2:** The partial characterization of agarases from different families.

Family	Bacterial Species and Protein	Accession No.	Optimal Temperature	Thermostability	*V*_max_ (μmol min^−1^mg^−1^)	Reference
GH16 (β-agarase)	*Catenovulum agarivorans* YM01^T^ (YM01-3)	AGU13985	60 °C	Stable below 50 °C for 1 h.	1.14 × 10^4^	This study
	*Agarivorans* sp. LQ48 (AgaA)	ACM50513	40 °C	Stable below 40 °C for 1 h.	909.1	[27]
	*Vibrio* sp. strain PO-303 (AgaA)	BAF62129	40 °C	Stable under 37 °C for 10 min.	22.5	[28]
	*Vibrio* sp. strain PO-303 (AgaD)	BAF34350	40 °C	Stable under 45 °C for 10 min.	101	[29]
	*Pseudoalteromonas* sp. CY24 (AgaA)	AAN39119	40 °C	Stable below 30 °C for 1 h.	482	[30]
	*Microbulbifer elongatus* JAMB-A7 (RagaA7)	BAC99022	50 °C	Stable up to 50 °C.	398	[31]
	*Microbulbifer* sp. Strain CMC-5 ( β-agarase)	None ^a^	50 °C	62% activity remained at 50 °C for 30 min.	0.133	[32]
	*Microbulbifer thermotolerans* JAMB-A94 (AgaA)	BAK08910	55 °C	Stable up to 60 °C.	517	[33]
	*Vibrio* sp. F-6 (AG-b)	None ^a^	55 °C	Stable below 60 °C for 30 min.	307.4	[16]
	*Pseudoalteromonas* sp. AG4 (AgaP)	ADD60418	55 °C	Stable below 55 °C for 1 h.	ND ^b^	[12]
GH50 (β-agarase)	*Agarivorans* sp. JA-1 (β-agarase)	ABK97391	40 °C	Stable up to 60 °C.	ND^ b^	[34]
	*Vibrio* sp. Strain CN41 (AgaACN41)	ADM25828	40 °C	Stable below 40 °C.	3	[35]
	*Agarivorans* sp. JAMB-AII (AgaAII)	BAD99519	40 °C	Stable up to 40 °C.	371	[36]
	*Streptomyces coelicolor* A3(2) (Sco3487)	CAB61811	40 °C	Stable below 50 °C for 1h.	10.75	[37]
	*Saccharophagus degradans* 2-40 (Aga50D)	ABD81904	30 °C	Stable up to 40 °C.	17.9	[38]
	*Alteromonas* sp. E-l (β-agarase)	BAE97587	40 °C	Stable up to 40 °C for 30 min.	ND^ b^	[4]
	*Agarivorans* albusYKW-34 (AgaA34)	P85974	40 °C	Stable up to 50 °C for 1 h.	529	[39]
	*Vibrio* sp. F-6 (AG-a)	None ^a^	40 °C	Stable below 50 °C for 30 min.	230	[16]
	*Agarivorans* sp. HZ105 (HZ2)	ADY17919	40 °C	ND ^b^	235	[40]
GH86 (β-agarase)	*Microbulbifer* sp. JAMB-A94 (rAgaO)	BAK08903	45 °C	Stable up to 40 °C.	ND^ b^	[41]
GH96 (α-agarase)	*Alteromonas agarilytica* GJB (AgaA)	AAF26838	42.5 °C	Stable up to 30 °C during for several days.	ND^ b^	[3]
	*Thalassomonas* sp. JAMB-A33 (AgaA33)	BAF44076	45 °C	Stable up to 40 °C for 30 min.	40.7	[42]
GH118 (β-agarase)	*Vibrio* sp. Strain PO-303 (Agarase C)	BAF03590	35 °C	Stable under 37 °C.	329	[43]
	*Pseudoalteromonas* sp. CY24 (AgaB)	AAQ56237	40 °C	Stable under 35 °C for 1 h.	ND^ b^	[44]
	*Catenovulum* sp. X3 (AgaXa)	GU975829	52 °C	Stable below 42 °C.	588.2	[45]

^a^ The accession No. is not available in the reference; ^b^ the characterization is not done.

Bacterial agarases are used in various preparative processes, namely, for liberating DNA and other embedded molecules from agarose gels, isolating protoplasts from red algae and generating neo-oligosaccharides, which exhibit important physiological and biological activities [46]. The industrial oligosaccharide production from agar or marine algae requires the agarases to have high activity and thermostability at temperatures higher than the gelling temperature of agar (around 40 °C) [41]. The use of thermostable agarase makes it possible to conduct the process at an elevated temperature, thereby considerably decreasing the risk of contamination, the cost of external cooling and enzyme dosage [47]. The elevated temperature also provides a better solubility of substrates and a lower viscosity, allowing accelerated mixing and pumping [48]. However, the optimal temperatures for most of the known agarases in the GH50 family were between 30 °C and 40 °C (Table 2). The β-agarase members of the GH86 and GH118 families exhibited the greatest activity at a temperature range of 35–52 °C, and the optimal temperatures of the two α-agarases were a little higher than 40 °C. Most of the agarases in the GH16 family had an optimal temperature around 40–50 °C. Although the agarase, YM01-3, showed similarity to other known agarases belonging to the GH16 family, whose highest optimal temperature for the agarolytic activity of the known agarases was 55 °C [12,16,33], the optimal temperature of the agarase, YM01-3, was 60 °C. Moreover, most of the characterized agarases were stable below 42 °C after incubation for 10 min to 1 h, while YM01-3, which was stable after incubation at 50 °C for 1 h, showed a higher thermostability than most of the other agarases, except AgaP from *Pseudoalteromonas* sp. AG4, AG-b from *Vibrio* sp. F-6, β-agarase from *Agarivorans* sp. JA-1 and AgaA from *Microbulbifer thermotolerans* JAMB-A94, which are stable up to 55–60 °C [12,16,33,34]. Some of the agarases showed the same thermostability with YM01-3, but lost most of the activity after incubation at 70 °C for 1 h [37,39]. However, YM01-3 maintained about 12% agarolytic activity, even if the recombinant agarase was boiled for 5 min. These characterizations revealed that the agarase, YM01-3, is thermostable, significantly different from the other reported agarases of GH16. Recently, another β-agarase gene, *agaXa*, was cloned from *Catenovulum* sp. X3, which is in the same genus as YM01^T^ [45]. AgaXa is also a thermostable agarase, but there are several differences between YM01-3 and AgaXa. YM01-3 showed very low identity to AgaXa (14.5%), and YM01-3 had maximum activity at 60 °C, while AgaXa was at 52 °C. In addition, YM01-3 was stable below 50 °C, while AgaXa was below 40 °C. Therefore, YM01-3 is more thermostable than AgaXa, and YM01-3 would provide more information for the thermostable mechanism and the structure-function relationship.

The thermostable mechanism of YM01-3 is not clear at present. It was reported that the fold state of the thermophilic protein showed a higher structural flexibility than that of the mesophilic homologue [49]. Compared with other agarases of GH16, YM01-3 owns less predicted parallel β-helixes (five helixes). Thus, it is assumed that a mechanism characterized by entropic stabilization could be responsible for the high thermostability of the enzyme. The three-dimensional structure would provide more information for the thermostable mechanism and the structure-function relationship; however, only four structural data of agarases are available to date, *i.e.*, three wild type β-agarases, AgaA, AgaB and AgaD, and a mutant, AgaB-E189D [24]. Further studies on the three-dimensional structure of β-agarase YM01-3 would provide not only a clearer understanding of its thermostable mechanism, but also additional data to other agarases from the GH16 family.

Since the superior limit of the stable range and optimal temperatures of YM01-3 are higher than the gelling temperature of agar (40 °C) and the *V*_max_ value (1.14 × 10^4^ U mg^−1^) is remarkably higher than any other agarases, the recombinant YM01-3 appears to be a powerful enzyme for use at low cost in industrial application with comparatively high agarolytic activity and thermostable characterization.

## 3. Experimental Section

### 3.1. Bacterial Strains and Growth Conditions

The marine bacterium, *Catenovulum agarivorans* gen. nov. sp. nov. YM01^T^ (=CGMCC 1.10245^T^ = DSM 23111^T^ = JCM 16580^T^) [21] was recently isolated from seawater of the Yellow Sea in the coastal region of Qingdao, China, and it was cultured at 28 °C on marine agar 2216 (MA) (Difco, Franklin lakes, NJ, USA). The pUCm-T (TaKaRa, Otsu, Japan) and plasmid pET24a (+) (Novagen, Madison, WI, USA) were used as the vector of cloning and expression, respectively. *Escherichia coli* JM109 (New England Biolabs, Beverly, MA, USA) and *E*. *coli* BL21 (DE3) (Novagen, Madison, WI, USA), which were used as the host for cloning and expression, were routinely grown at 37 °C in Luria-Bertani (Difco, Franklin lakes, NJ, USA) broth supplemented with ampicillin or kanamycin (100 μg mL^−1^) when required.

### 3.2. Analysis of the Agarolytic Activity of the Extracellular Proteins of YM01^T^

The broth culture of YM01^T^ was centrifuged at 12,000 rpm for 15 min at 4 °C to remove the cells. Solid ammonium sulfate was added to the supernatant to 60% saturation at 4 °C. Precipitated proteins were pelleted by centrifugation (12,000 rpm for 15 min, 4 °C), resuspended in PBS (0.01 M, pH 7.2) and then dialyzed in the same buffer for 24 h at 4 °C. The SDS-PAGE was performed, and the gel was overlaid onto a plate sheet containing 2% agarose and incubated at 37 °C for 3 h. Following incubation, the agarose sheets were stained by 2% (w/v) iodine solution, and then, agarase activity was visualized as clear zones on a brown background; meanwhile, protein bands were visualized by staining with Coomassie Brilliant Blue R-250 [42]. Finally, by comparing the zymogram and the gel, the gel band that contained the protein with the highest agarase activity was sliced, washed and then analyzed by mass spectrometry (Bo-Yuan Biological Technology Co., LTD, Shanghai, China).

### 3.3. Cloning and Sequence Analysis of the YM01-3 Gene

The YM01-3 gene (GenBank accession No. KF413621) was amplified by polymerase chain reaction (PCR) using the primer sets, YMa (5′-CCGGAATTCATGTATGCAGCAGACTGGGAT-3′) and YMb (5′-CCGCTCGAGTTGGAACTTCCATTGCTGG-3′), which contained *EcoR*I and *Xhol*I sites (underline), respectively. The termination codon (TAA) was removed in the reverse primer in order to add His-tag to the C-terminal of the recombinant agarase. The PCR products with an appropriate size were gel purified and ligated into the pUCm-T vector. The plasmid was transformed into *E*. *coli* JM109 competent cells, and the cloned gene was confirmed by sequencing. The recombinant plasmid, pUCm-T/YM01-3, was digested by *EcoR*I and *Xhol*I restriction endonucleases, and the product was purified and inserted into the linearized plasmid, pET-24a (+).

The prediction of the amino acid sequence was performed with the software, BioEdit (Ibis Biosciences, Carlsbad, CA, USA). The signal peptide was predicted using the SignalP 4.0 server [23]. Homology searches were performed using BLASTP [50,51]. Multiple alignments of protein sequences from different species were performed by ClustalW [52,53]. The phylogenetic tree based on the amino acid sequence was constructed by MEGA5.02 [54].

### 3.4. Overexpression and Purification of the Recombinant YM01-3 Protein

The expression vector, pET-24a (+)/YM01-3, was transformed into *E*. *coli* JM109 competent cells, and correct recombinants were selected after sequencing confirmation. The sequence confirmed that pET-24a (+)/YM01-3 was transformed into *E*. *coli* BL21 (DE3) cells and grown at 37 °C in LB broth supplemented with kanamycin (100 μg mL^−1^). At the mid-exponential growth phase, IPTG was added at a final concentration of 0.1 mM, and the cultivation was continued further at 16 °C, 150 rpm, for 10–12 h.

The induced *E*. *coli* cells were collected by centrifugation, and the His-tagged agarase was purified using the His Bind Kit, according to the recommendations of the manual. The purified agarase YM01-3 was dialyzed and freeze-dried for determination of molecular mass by mass spectroscopy (Thermo Scientific LTQ Orbitrap XL, Waltham, MA, USA).

### 3.5. Agarolytic Activity Assay of the Recombinant YM01-3

The agarolytic activity of purified recombinant YM01-3 was assayed by the modified 3,5-dinitrosalicylic acid method [27]. Briefly, the standard reaction contained 100 μL of diluted enzyme solution and 900 μL of 0.1 M citric acid-sodium citrate buffer (pH 6.0) containing 0.25% (w/v) agarose as the substrate. After incubation at 60 °C for 10 min, 1 mL of 3,5-dinitrosalicylic acid was added to the mixture and boiled for 5 min until the color developed. The reaction was terminated by cooling the test tube in a cold water bath. The absorbance of reducing sugar was measured at 540 nm and compared to the standard curve of d-galactose [45]. Enzyme activity (U) was defined as the amount of enzyme that liberated 1 μmol of d-galactose per minute.

### 3.6. Biochemical Characterization of the Recombinant YM01-3

The optimal temperature of the recombinant agarase was determined by monitoring the relative activity at temperatures ranging from 40 to 100 °C (at 10 °C intervals) in 20 mM Tris-HCl buffer (pH 8.0) for 10 min. The thermostability of the protein was determined by measuring the residual activity of the enzyme after incubation at temperatures ranging from 0 to 100 °C for 1 h. The relative activity was defined as the percentage of activity determined with respect to the maximum agarolytic activity. 

The optimal pH of the agarase was assayed with a pH range of 4.0–11.0 (at 1.0 intervals) at 50 °C. The buffers used were 0.1 M citric acid/sodium citrate buffer (pH 4.0–6.0), 20 mM Tris-HCl buffer (pH 7.0–8.0), 50 mM Tris-glycine buffer (pH 9.0–10.0) and 50 mM Na_2_HPO_4_-NaOH buffer (pH 11.0). The pH stability of the agarase was determined by pre-incubating the enzyme in buffers with a pH range of 4.0–11.0 at 4 °C for 12 h, and the residual enzymatic activity was measured.

The sensitivity of YM01-3 to various metal ions, denaturants and reducing agents was determined by the measurement of activity after incubation in 1% agarose solution supplemented with different ions (1 mM) and agents (10 mM) at 45 °C for 30 min. *K*_m_ and *V*_max_ values for agarase acting on agarose (final concentration of 1–5 mg mL^−1^) were calculated by linear regression analysis of Lineweaver-Burk double reciprocal plots of initial velocity data obtained under the standard condition described above.

### 3.7. Identification of Hydrolysis Products by Purified YM01-3

The hydrolysis reactions were conducted at 50 °C in 20 mM Tris-HCl buffer (pH 8.0) containing the purified YM01-3 agarase and 0.25% agarose. Subsequently, 1 mL of the reaction solution was spotted onto Silica Gel 60 TLC plates (Merck, Germany), which were developed using a solvent system composed of *n*-butanol–acetic acid–water (2:1:1, v:v:v). The oligosaccharides spots that resulted from hydrolysis of substrates were visualized after the plate was sprayed with a modified diphenylamine-aniline reagent and heated at 100 °C for 10 min [30].

To identify the agarose degradation products by YM01-3 agarase, the reaction mixtures after incubation for 1 h were applied to Silica Gel 60 TLC plates. Three quarters of the plate was covered, and the remainder was sprayed and heated, as mentioned above, for visualization. According to the position of the spots in the visualized part, the spots corresponding to the hydrolyzed products were scraped out with a razor from the TLC plate without diphenylamine-aniline reagent treatment and dissolved in the mixture of hexanenitrile and 1 mM NH_4_HCO_3_ (1:1, v:v). The molecular mass distribution of the products was determined by ion trap mass spectrometer (Thermo Scientific LTQ Orbitrap XL, Waltham, MA, USA).

### 3.8. Statistical Analysis

Multiple comparisons were performed by the one-way analysis of variance (ANOVA) using the Tukey test (SPSS statistics 19.0 software, IBM, Armonk, NY, USA). Results were expressed as the mean ± SEM from three independent experiments, with *p* < 0.05 and *p* < 0.01 taken to show statistical significance and distinct significance, respectively.

## 4. Conclusions

The present study represents cloning and overproducing of YM01-3, which showed the most evident activity on agar degradation among the 15 putative agarases from *Catenovulum agarivorans* YM01^T^. The recombinant YM01-3 reveals high agar degrading activity and produces neoagarotetraose and neoagarohexaose as the main products. Notably, YM01-3 shows higher optimal temperature and thermostability than most of the other agarases, which reveals its potentially high effectiveness for application in industrial oligosaccharide production.
